# Making fish oils in plants: from alpha to omega

**DOI:** 10.1111/nph.71292

**Published:** 2026-05-26

**Authors:** Johnathan A. Napier

**Affiliations:** ^1^ Rothamsted Research Harpenden AL5 2JQ UK

**Keywords:** biotechnology, nutritional enhancement, omega‐3, transgenic, translation & impact

## Abstract

The transgenic accumulation of omega‐3 long‐chain polyunsaturated fatty acids represents one of the most complex assemblies of heterologous sequences in a plant host. Through the combined efforts of multiple research teams from public and private sectors, GM crops synthesising these important fatty acids have progressed from the initial proof‐of‐principle stage to fully regulated and approved products. That this has taken over 25 years should not be surprising, but equally, there are several aspects of the sophisticated metabolic engineering which remain unresolved. The aim of this short review is to shine a light on some of the less familiar aspects of these efforts and to maybe prompt renewed consideration of the associated issues. In particular, the oft‐ignored topic of transgene silencing in plant biotechnology is considered, as is the potential of multicistronic polyprotein fusions to reduce construct complexity and sequence repetition. Collectively, it is hoped that focussing on these important but neglected topics will inspire a new wave of research activity, underpinning a further shift to a fully sustainable bioeconomy.

## Graphical abstract

Schematic representation of the biosynthetic pathway for the synthesis of omega‐3 long‐chain polyunsaturated fatty acids. The sequential transgene‐encoded activities required to convert endogenous fatty acids to the longer chain polyunsaturated forms of eicosapentaenoic acid and docosahexaenoic acid are shown in red. The metabolic bottleneck (‘substrate dichotomy’) arising from the different substrate preferences of desaturases and elongases is also represented. Transgene‐encoded activities are predominantly sourced from marine microalgae but also from oomycetes and bryophytes.

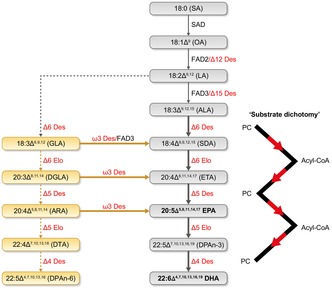

**Table:** 

	Contents	
	Summary	1013
I.	Introduction	1013
II.	Metabolic engineering in the analogue age	1014
III.	Foundational studies in the transgenic accumulation of nonnative fatty acids	1014
IV.	After the genetic Goldrush	1014
V.	Extending the pathway to DHA	1015
VI.	Nature knows best	1016
VII.	The heart of darkness – epigenetics in biotechnology	1016
VIII.	Conclusions and future prospects	1017
	Acknowledgements	1017
	References	1017

## I. Introduction

It is now over 20 years since the first reports of the transgenic accumulation of omega‐3 long‐chain polyunsaturated fatty acids (LC‐PUFA) were published, and this serves as a suitable milestone to consider what has been achieved in the field of plant lipid biotechnology and what remains to be resolved. Fatty acids such as eicosapentaenoic acid (EPA) and docosahexaenoic acid (DHA) have long been identified as desirable target compounds for heterologous synthesis in transgenic plants (Damude & Kinney, 2008). This is because omega‐3 long‐chain polyunsaturated fatty acids such as EPA and DHA are nonnative to higher plants but have considerable value and utility, specifically in terms of cardiovascular health benefits and anti‐inflammatory properties, also as a key component of aquafeed diets (Venegas‐Calerón & Napier, 2023) not least of all since marine sources of these fatty acids can no longer meet global demand (Tocher *et al*., 2019). Thus, once it became apparent via the pioneering work of the Calgene company that it was possible to modify and enhance the fatty acid composition of seed oils (Voelker *et al*., 1992), much attention was directed towards trying to engineer plants to accumulate EPA and DHA. However, as those of us who have been involved in this can testify, this was by no means easy or straightforward (Napier, 2025), and the (significant) achievements have only been obtained through protracted and focussed effort. It is timely to consider what still remains to be elucidated, in terms of the primary biosynthesis, and also how new approaches might advance our understanding.

## II. Metabolic engineering in the analogue age

Although it is not the purpose of this article to review the many stages on the journey to successfully engineer plants to accumulate omega‐3 LC‐PUFAs, it is worth considering what was achieved with the tools available at the time. There is a slightly mischievous school of thought which likes to suggest that before the more recent advent of synthetic/engineering biology, metabolic engineering was, at least in the plant sector, something akin to banging rocks together to discover fire. However, this is certainly not the case for plant lipid engineering, with pathway engineering using up to nine independently expressed genes being reported over 20 years ago (Wu *et al*., 2005). And on perhaps a more serious note, it is important to remember that most of the resources that we currently routinely deploy did not exist or were in their infancy at the turn of the century. For example, gene synthesis was prohibitively expensive, whole‐genome sequencing was a major undertaking, and most cloning was via type II restriction digestion and ligation, meaning that multigene engineering was a complicated and sometimes painful experience. But despite all these challenges, some remarkable and impressive advances were achieved, indicating that while tools can improve, ingenuity and drive are also key ingredients for success.

## III. Foundational studies in the transgenic accumulation of nonnative fatty acids

**Fig. 1:**
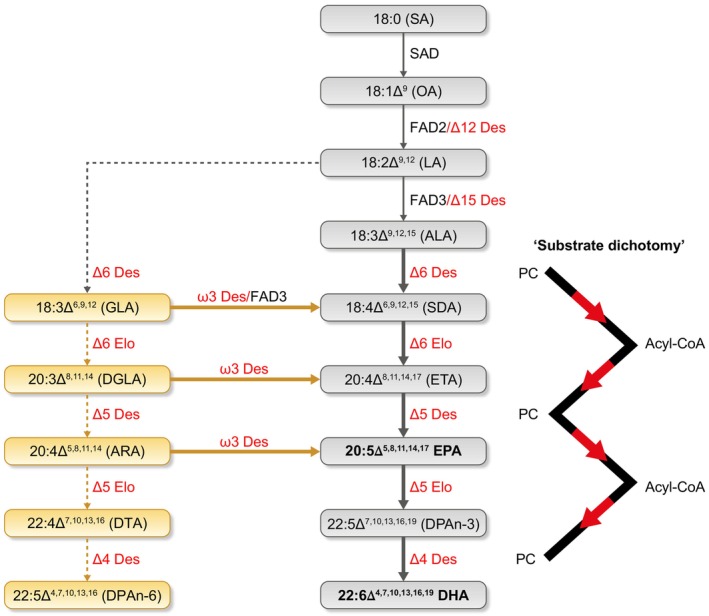
Schematic representation of the biosynthetic pathway for the synthesis of omega‐3 long‐chain polyunsaturated fatty acids. The sequential transgene‐encoded activities required to convert endogenous fatty acids to the longer chain polyunsaturated forms of eicosapentaenoic acid (EPA) and docosahexaenoic acid (DHA) are shown in red. The metabolic bottleneck (‘substrate dichotomy’) arising from the different substrate preferences of desaturases and elongases is also represented. Transgene‐encoded activities are predominantly sourced not only from marine microalgae but also from oomycetes and bryophytes.

The biosynthetic pathway for omega‐3 LC‐PUFAs requires the conversion of endogenous (native) C18 fatty acids into longer chain forms (C20–22) that also contain additional double bonds (hence the name ‘polyunsaturated’). This conversion is catalysed by the sequential and alternating actions of acyl‐desaturases (adding double bonds at specific positions on the acyl chain) and acyl‐elongases (adding two carbons to the carboxyl end of the fatty acid), and these two types of reactions are carried out by substrate‐specific enzymes (Ruiz‐Lopez *et al*., 2015b). In the case of the desaturation reaction, this is catalysed by an unusual variant form of microsomal desaturase, with a cytochrome b_5_ domain present at the N‐terminus. It has been hypothesised that this configuration is related to the specific ‘front‐end’ desaturation that is carried out during the biosynthesis of EPA and DHA, and it is the case that disruption of the b_5_ domain results in a nonfunctional enzyme which cannot be rescued by microsomal cytochrome b_5_ working in *trans*. Perhaps more importantly, the N‐terminal cytochrome b_5_ domain serves as a diagnostic motif for the identification of desaturases involved in the biosynthesis of omega‐3 LC‐PUFAs, as all the known examples of PUFA desaturases contain this motif, which is otherwise absent from other microsomal desaturases (Venegas‐Calerón & Napier, 2023). However, beyond being essential for enzyme function, it remains to be clarified why these desaturases require a physically linked electron donor. Equally, the evolution of PUFA desaturases is obscure, although a study of the nematode *Caenorhabditis elegans* (which synthesises EPA) genome indicates that the genes that encode the PUFA desaturases have arisen as a result of gene duplication and subsequent divergence (Napier & Michaelson, 2001; Watts & Browse, 2002). Specifically, the Δ6‐ and Δ5‐desaturases (which catalyse the sequential desaturation reactions required for the synthesis of EPA – see Fig. 1) are present in a diverse range of taxonomically unrelated organisms, including Stramenopiles, Chlorophyta, and other aquatic unicellular organisms, but also extending to some oomycetes and invertebrates. In general, it is considered that marine microbes form the base of the ‘omega‐3 food web’, as these highly abundant flora and fauna represent the primary source of these fatty acids in the natural trophic systems (Napier & Betancor, 2023). Interestingly, not all marine algae synthesise omega‐3 LC‐PUFAs (Lang *et al*., 2011), so it is clearly not an obligate requirement for form or function. The evolutionary basis for the creation and retention of the capacity to synthesise EPA and DHA is equally obscure, and a better understanding of this might further enhance metabolic engineering strategies to engineer a heterologous host with the capacity to synthesise these nonnative fatty acids. Certainly, it is striking that no Angiosperm plant has been identified with the capacity to synthesise EPA or DHA, whereas synthesis of EPA is common (but not essential) in Bryophytes.

## IV. After the genetic Goldrush

Around the end of the last millennium, with the advent of new DNA‐sequencing methods, gene identification became significantly more routine. Whole‐genome and transcriptome analysis provided straightforward methods to identify genes of interests from any given organism. In the case of the synthesis of omega‐3 LC‐PUFAs, culture collections were characterised for the abundance of these fatty acids and sequenced accordingly (Lang *et al*., 2011). This led to a relatively large ‘toolbox’ of potential sequences from which to attempt to reconstitute the heterologous synthesis of EPA and DHA, although as many of these efforts were carried out by private industry, these sequences were subject to IP restrictions. Irrespective of that, by 2003, multiple examples of both desaturase and elongases for the synthesis of EPA or DHA had been cloned and characterised, along with the demonstration that it was feasible to engineer the accumulation of their product fatty acids in a taxonomically distinct host such as yeast (Napier *et al*., 2003).

Two foundational studies were published in 2004 (Abbadi *et al*., 2004; Qi *et al*., 2004) which demonstrated the feasibility of making omega‐3 LC‐PUFAs such as EPA in transgenic plants. The two studies opted to use different configurations of the biosynthetic pathway and also used different promoters (constitutive vs seed‐specific), but both were recognised as being significant advances in the field of plant lipid research and wider plant biotechnology. Perhaps more importantly, these studies highlighted some of the gaps in our understanding of lipid metabolism and how to manipulate it in a predictive fashion. For example, Abbadi *et al*. (2004) observed the significant build‐up of biosynthetic intermediates, specifically the C18 Δ6‐desaturated fatty acids produced by the initiating reaction of the heterologous pathway. These nonnative fatty acids accumulated to very high levels, and unlike the situation with other nonnative fatty acids such as ricinoleic acid (Bates *et al*., 2014), were clearly not subject to beta‐oxidation or acyl‐editing. This so‐called substrate dichotomy bottleneck took additional effort to resolve but was identified as the consequence of nonnative biosynthetic enzymes using acyl substrates from different metabolic pools, specifically acyl‐CoAs vs phospholipid‐linked fatty acids (Fig. 1). Interestingly, the alternative configuration of the biosynthetic pathway adopted by Qi *et al*. (2004) serendipitously bypassed the substrate dichotomy bottleneck, and for some time, it was the more favoured route for transgenic synthesis of EPA (Damude *et al*., 2008; Petrie *et al*., 2010a,b; Ruiz‐Lopez *et al*., 2015a). However, additional challenges were observed, such as enzyme promiscuity and also flux of acyl‐chains into the pathway, which refocussed efforts on the Δ6‐pathway.

## V. Extending the pathway to DHA

The demonstration that it was possible to make the C20 omega‐3 EPA was quickly followed by reports showing the successful synthesis of the C22 DHA. Since marine‐derived fish oils usually contain both these fatty acids, it was logical to attempt to replicate this in a terrestrial‐produced alternative. Marine fish oils usually contain either *c*. 20% EPA and DHA (Northern hemisphere oil) or 30% EPA and DHA (Southern hemisphere oils), reflecting their different diets and associated trophic inputs (Napier & Betancor, 2023). However, unlike the relatively successful ‘proof of principle’ results obtained for EPA, the amount of DHA was significantly lower. For example, Robert *et al*. (2005) reported up to 0.5% DHA in the seed fatty acids of transgenic Arabidopsis. Similarly, Wu *et al*. (2005) reported the accumulation of 0.2% DHA in the seed fatty acids of transgenic *Brassica juncea*. This latter example was achieved via the seed‐specific expression of nine different transgenes encoding the five genes required for the synthesis of DHA plus additional activities to improve flux of substrates, and to this day represents one of the most complex examples of transgenic plant metabolic engineering successfully undertaken. Equally impressive as a feat of transgenic engineering was the expression of the microalgal PKS‐like PUFA synthase complex in transgenic canola (Walsh *et al*., 2016), although this large (*c*. 30 Kb) ORF proved cumbersome to optimise. Both Wu *et al*. (2005) and Walsh *et al*. (2016) utilised a multisite Gateway recombination system to assemble the final construct for transformation and we have also successfully built large multigene constructs with the same approach (Ruiz‐Lopez *et al*., 2014). Other assembly rationales have been applied to this pathway, including *de novo* synthesis (Petrie *et al*., 2020). However, the desirability of being able to swap or modify the 30+ parts that comprise these constructs constrains the approaches available.

While these initial levels of DHA represented amounts that were at least 20‐fold less than would be required for a drop‐in replacement of fish oil, they provided the basis for iterative improvement and design optimisation (analogous to the *Design‐Build‐Test‐Learn* mantra subsequently adopted by the synthetic biology community). The earlier study by Abbadi *et al*. (2004) had highlighted the importance of utilising the acyl‐CoA pool to bypass phospholipid‐dependent processes and the associated substrate dichotomy (Fig. 1), and this triggered a search for PUFA (front‐end) desaturases that had a preference for acyl‐CoA substrates as opposed to the more prevalent phospholipid‐dependent form. Petrie *et al*. (2010a,b) identified an acyl‐CoA‐dependent Δ6‐desaturase from *Micromonas pusilla*, and Hoffmann *et al*. (2008) reported the characterisation of acyl‐CoA‐dependent desaturases from *Mantionella squamata*. Sayanova *et al*. (2012) used advanced lipidomics to demonstrate the acyl‐CoA substrate preferences of multiple desaturases, including the Δ6‐desaturase from *Ostreococcus tauri*, which was serendipitously identified during an algal genome sequencing project (Domergue *et al*., 2005) and observed to be a highly active enzyme. Although acyl‐CoA‐dependent desaturases are considered to be the predominant form of these enzymes in vertebrates, most desaturases in plants and algae are phospholipid‐dependent (i.e. utilizing acyl substrates that are contained in membrane lipids). It is therefore rather surprising that acyl‐CoA‐dependent desaturases were identified from the order Mamiellales, in the class Mamiellophyceae of green algae. There is no obvious evolutionary explanation for the occurrence of such enzyme activities in these species (Li *et al*., 2020), and this is made more remarkable given the highly compact genomes of these so‐called picoalgae. Irrespective of that, acyl‐CoA Δ6‐desaturases from this clade have proved key to the efficient reconstitution of the synthesis of EPA and DHA in transgenic plants. There is no experimental evidence that other (Δ5, Δ4) desaturases from the biosynthetic pathway (Fig. 1) share the same acyl‐CoA substrates‐specificity as the Δ6‐desaturase, which is equally perplexing from a functional perspective. It is hoped that in the absence of any protein structure for these enzymes, tools such as AlphaFold can shed light based on this acyl‐CoA substrate preference and if it could be engineered into other desaturases.

## VI. Nature knows best

The observation that natural variation in how different organisms configure enzymes and pathways to synthesise EPA and DHA, combined with significant decreases in the cost and availability of genome and transcriptome sequences, prompted further closer examination of many PUFA‐synthesising species with the goal of identifying superior biosynthetic activities. As well as the precedent of the acyl‐CoA Δ6‐desaturase, we previously identified phospholipid‐dependent Δ6‐desaturases from Primula species, which showed varying preference for either omega‐3 or omega‐6 substrates – this indicated the possibility of enhancing flux via substrate preference (Sayanova *et al*., 2003). Other examples include bifunctional w3 and w6‐desaturases that have activity towards both forms of acyl substrates, identified from *Fusarium moniliforme* (Damude *et al*., 2006) and from the protozoa *Acanthamoeba castellanii* (Sayanova *et al*., 2006). These activities were demonstrated as directing optimal synthesis of EPA in various heterologous systems such as yeast, but the benefit of combining such activities with the primary biosynthetic enzymes remains to be demonstrated in transgenic plants and may yet prove a useful addition to current configurations of the pathway.

A further interesting and underexplored configuration of the biosynthesis of EPA and DHA is based around another serendipitous discovery that the biosynthetic activities for the DHA module (the Δ5‐elongase and the Δ4‐desaturase; Fig. 1) existed as a translational fusion in algal species such as *Euglena gracilis* (Damude *et al*., 2008). Intuitively, there are some potential benefits for sequential activities in a biosynthetic pathway to be in physical proximity (as a consequence of a noncleavable linker; cf the cytochrome b5 fusion domain of the PUFA desaturases). However, since (as discussed above) the desaturase and elongase activities are accessing different metabolic pools (phospholipids vs acyl‐CoA), it could be argued that such proximity is unnecessary unless these two substrate pools are co‐localised. Another open question could be if the fused form of the DHA module was more efficient, why did it not evolve more often? Currently, the use of noncleaved linkers to enhance flux through the omega‐3 LC‐PUFA biosynthetic pathway has not been widely exploited in transgenic plants but may represent an opportunity for further improvement. Equally, the use of ‘self‐cleaving’ (ribosome‐skipping) linkers such as the 2A peptide (Halpin *et al*., 1999) might represent an approach to reduce the number of expression cassettes required to produce the target compounds – such systems are routinely used for the RUBY marker which comprises three activities but is expressed as a single transcript and also recently used in the seed specific accumulation of the ketocarotenoid astaxanthin (Jeong *et al*., 2021).

## VII. The heart of darkness – epigenetics in biotechnology

Although it is now over 35 years since the first reports of the phenomena we now know as (trans)gene‐mediated silencing (Matzke *et al*., 1989), there are relatively few overt recognitions in the academic literature of the impact that epigenetics plays on plant biotechnology, especially for complex multigene traits of the type described here. Almost certainly, many occurrences of transgene silencing are simply discarded, most likely based on poor accumulation of the target compounds, complicating our ability to determine the functionality of a particular transgene configuration and/or its susceptibility to these epigenetic processes. In the case of the omega‐3 LC‐PUFA pathway, where multiple nonnative sequences are expressed often from the same promoter, it might not be surprising to detect transgene silencing. We have observed strong silencing of one of our constructs, although this was limited to only one cassette in the construct and could be related to proximity to the T‐DNA border (Han *et al*., 2022). Equally, Naim *et al*. (2015) reported the use of the P19 viral silencing‐suppressor protein from *Tomato bushy stunt virus* to prevent transgene silencing of the LC‐PUFA pathway in Arabidopsis. In their study, Naim *et al*. (2015) used seed‐specific expression of the P19 protein to avoid some of the consequences of interfering with normal plant development (arising from perturbing endogenous microRNA metabolism) and reported increased accumulation of target C20 PUFAs such as arachidonic acid. It remains an open question as to whether using targeted expression of P19 would be an effective intervention for controlling transgene silencing in polyploid crops such as camelina and canola, and crucially, it would be unlikely to be capable of reversing silencing. Alternative approaches could include targeting RNA‐dependent RNA polymerase RDR6 that plays a key role in generating templates for the DICER activities which ‘programme’ the sequence‐specific silencing pathways – RDR6 has been shown to play a role in the silencing of the FAD2 desaturase (Chen *et al*., 2023). Recently, targeted expression of RDR6 in a *rdr6* mutant background allowed for the tissue‐specific suppression of the pleiotropic effects associated with this mutation as well as the suppression of silencing of transgenic FAD2 in the target seed tissue (Li *et al*., 2025). Certainly, as constructs become even more complex and integrations become larger (as envisaged with many Engineering Biology projects), the issues of transgene silencing will become more pressing. In the case of traits such as the omega‐3 LC‐PUFAs, where the ultimate goal is commercial cultivation at large scale, the avoidance or mitigation of transgene silencing is critical, especially once a lead event embarks on the expensive pathway to regulatory approval (Napier, 2025).

## VIII. Conclusions and future prospects

Although a complex multigene trait, the transgenic production of omega‐3 LC‐PUFAs such as EPA and DHA is an exemplar of the power of plant biotechnology to deliver useful traits capable of improving nutrition and mitigating environmental degradation. Over the course of 25 years, research from multiple teams, in academia and industry, has converged on delivering a validated, sustainable alternative to marine extraction of fish oils (Ruiz‐Lopez *et al*., 2014; Petrie *et al*., 2020). It is often (erroneously) suggested that GM has failed to deliver the second generation of products, specifically those output traits focussed on consumer benefit and dietary health. However, in the case of EPA and DHA, that is happily not the case – products have been through regulatory approval (Suh *et al*., 2023) and are now on the market (MacIntosh *et al*., 2021), with further improvements in the pipeline (Napier, 2025). While it could be argued that this success is hardly overnight, it likely is a true reflection on the decadal timescales that are associated with developing and deploying a truly ground‐breaking innovation in the (literal) field of plant biotechnology.

## Competing interests

None declared.

## Disclaimer

The New Phytologist Foundation remains neutral with regard to jurisdictional claims in maps and in any institutional affiliations.
